# Halophilic archaea as tools for bioremediation technologies

**DOI:** 10.1007/s00253-024-13241-z

**Published:** 2024-06-29

**Authors:** Rosa María Martínez-Espinosa

**Affiliations:** 1 Biochemistry and Molecular Biology and Edaphology and Agricultural Chemistry Department, Faculty of Sciences, University of Alicante, Ap. 99, E-03080 Alicante, Spain; 2 Multidisciplinary Institute for Environmental Studies “Ramón Margalef”, University of Alicante, Ap. 99, E-03080 Alicante, Spain

**Keywords:** Haloarchaea, Bioremediation, Green biotechnology, Heavy metals, Hydrocarbons, Nanoparticles, Wastewater treatments

## Abstract

**Abstract:**

Haloarchaea are extremophilic microorganisms belonging to the Archaea domain that require high salt concentrations to be alive, thus inhabiting ecosystems like salty ponds, salty marshes, or extremely salty lagoons. They are more abundantly and widely distributed worldwide than initially expected. Most of them are grouped into two families: Halobacteriaceae and Haloferacaceae. The extreme conditions under which haloarchaea survive contribute to their metabolic and molecular adaptations, thus making them good candidates for the design of bioremediation strategies to treat brines, salty water, and saline soils contaminated with toxic compounds such as nitrate, nitrite, oxychlorates such as perchlorate and chlorate, heavy metals, hydrocarbons, and aromatic compounds. New advances in understanding haloarchaea physiology, metabolism, biochemistry, and molecular biology suggest that biochemical pathways related to nitrogen and carbon, metals, hydrocarbons, or aromatic compounds can be used for bioremediation proposals. This review analyses the novelty of the most recent results showing the capability of some haloarchaeal species to assimilate, modify, or degrade toxic compounds for most living beings. Several examples of the role of these microorganisms in the treatment of polluted brine or salty soils are also discussed in connection with circular economy-based processes.

**Key points:**

• *Haloarchaea are extremophilic microorganisms showing genuine metabolism*

• *Haloarchaea can metabolise compounds that are highly toxic to most living beings*

• *These metabolic capabilities are useful for designing soil and water bioremediation strategies*

## Introduction

Global pollution is one of the main current concerns worldwide due to the negative impact on air, soil/water quality, and the decrease in biodiversity and living wellness (Breisha and Winter 2010; Le Borgne et al. 2008). For example, based on data from the World Health Organization, almost all of the global population (99%) are exposed to air pollution levels that put them at increased risk for diseases including heart disease, stroke, chronic obstructive pulmonary disease, cancer, and pneumonia (https://www.who.int/data/gho/data/themes/air-pollution). Regarding water, data from the United Nations stated that 2.2 billion people still lack access to safely managed drinking water services. Around 80% of wastewater flows back into the ecosystem without being treated or reused (https://www.un.org/en/global-issues/water).

Apart from concerns regarding the emission of greenhouses and the consequent issues related to global climate change, the significant presence of petroleum derivatives, heavy metals, xenobiotic compounds, and micro and nano plastics (among other contaminants) in the environment is currently included in the political and environmental management agendas of many countries and has focused the attention of the scientific community intending to find solutions that mitigate the release of these pollutants and remedy the negative environmental impact already caused by their presence in soil, water, and even air (Goyal et al. 2023; Yuan et al. 2024). Although global pollution is affecting different types of ecosystems indiscriminately, recent literature and databases suggest that aquatic systems and soils are probably the most affected media during the last few years (Breisha and Winter 2010; Le Borgne et al. 2008; Goyal et al. 2023; Yuan et al. 2024).

There are many physicochemical and biological methods described by the literature aiming at the removal of pollutants that have been used successfully, mainly during the last three decades. More recently, biological-based methods have received increasing attention as a way of removing or neutralising contaminants from a contaminated site using native or introduced plants and/or microorganisms (Azubuike et al. 2016). Among biological approaches focusing attention worldwide, bioremediation is addressing several innovative approaches. Bioremediation is defined as the application of the metabolic capabilities of microorganisms (including bacteria, some algae, yeast, and fungi) as well as plants to degrade and/or assimilate compounds that are toxic to most living organisms or to transform them into less toxic substances. Some living beings are even able to bioassimilate those pollutants by naturally enhancing metabolic pathways that allow the degradation or assimilation of those compounds (Azubuike et al. 2016; El Fantroussi and Agathos 2005). In this context, two main types of bioremediation strategies might be considered according to the use of external supplements:

i) Intrinsic *in situ* bioremediation: Natural degradation of remaining pollutants in the environment, without any external intervention
ii) Engineered *in situ* bioremediation: Which requires an external supply to increase the biodegradation rate by accelerating the growth of organisms, thanks to the secretion of essential nutrients

A deep analysis of the reported literature reveals that more than 1000 patents have been granted to different technical in situ bioremediation aspects, and engineered bioremediation prevails over the intrinsic one except for those cases in which the natural occurrence of biodegradation is a fast process (Wang et al. 2021).

Those strategies could be improved through engineered bioremediation which is mainly made utilising two approaches: biostimulation (implies the addition of limiting nutrients to a polluted environment in which all the necessary natural microorganisms are assumed to be present and metabolically active) and bioaugmentation (considers the monitoring of parameters like temperature and pH to guarantee that their values allow optimised microbial growth and metabolism) (El Fantroussi and Agathos 2005, Wang et al. 2021). Bioaugmentation oversees and guarantees the quantity and diversity of microorganisms showing the desired catalytic capability needed for biodegradation of specific compounds (Adams et al. 2015; Cycoń et al. 2017; Muter 2023). A combination of these approaches is suggested to be a promising strategy to speed up bioremediation (El Fantroussi and Agathos 2005). These strategies can be improved by using modified microbial strains whose metabolic capabilities are enhanced to make more efficient the removal of pollutants.

In summary, bioremediation is considered one of the best options to decontaminate polluted environments due to its significant number of advantages such as in situ treatment of soils or waters, high efficiency, low cost in most cases, and no secondary pollution (El Fantroussi and Agathos 2005; Adams et al. 2015; Cycoń et al. 2017).

This review summarises the main advances in bioremediation with special emphasis on the use of microbial species of haloarchaea, a group of extremophilic microorganisms characterised by their high tolerance to compounds that are extremely toxic for most living beings.

## Microorganisms for bioremediation

Phytoremediation was first defined as an efficient approach to degrade contaminants mainly from soil sediments and groundwaters using plants and associated soil microbes (Kafle et al. 2022). It is considered a cost-effective and environmentally sound approach, which uses plants to immobilise, stabilise, extract, degrade, or reduce the toxicity of contaminants. However, recent analysis of the research done in this area suggests that phytoremediation is the most suitable bioremediation approach only for remote regions with low land values because since these regions allow a longer period to be restored, land vegetation covers can be established in a reasonable period like natural attenuation. Considering that the length of phytoremediation is an inherent limitation, this disadvantage limits its implementation in developed regions, such as growing urban areas. Because high land values could not be recovered in the short term, phytoremediation is not cost-effective in those regions (Wang and Delavar 2023).

For this reason, during the last decades, phytoremediation techniques have been improved by incorporating microbial consortia, such as phosphate-solubilising microbes, to achieve a most trustworthy approach for the enhancement of the remediation (Jia et al. 2016). Microbial biodiversity on a global scale is incalculable even today. Microorganisms are ubiquitous, show a wide range of metabolic capabilities, and can use a wide range of substrates as carbon and nitrogen sources; hence, they are found in unusual environments where they can absorb a wide range of pollutants. Consequently, microorganisms have become great tools for the design and implementation of bioremediation approaches themselves (as microbial consortia) or in consortia with plants (Cao et al. 2022; Wu et al. 2023). Microorganisms can remove a significant range of pollutants from the environment, thanks to different mechanisms; all of them mainly grouped into two broad categories: immobilisation and mobilisation. The mobilisation process includes enzymatic oxidation, bioleaching, biostimulation, bioaugmentation, and enzymatic reduction procedure, whilst the immobilisation includes bioaccumulation, complexation, biosorption, and precipitation (solidification) (Jia et al. 2016; Cao et al. 2022; Wang and Delavar 2023; Wu et al. 2023). Microorganisms carrying out mineralisation can transform pollutants into end products such as carbon dioxide and water or other intermediate metabolic substances. During immobilisation, several microbial species can convert compounds into a form that will be unavailable in the environment. Immobilisation processes can be used as tools for the removal of contaminants using both the *in situ* and the *ex situ* methods (Jia et al. 2016; Kafle et al. 2022; Wang and Delavar 2023). The *ex situ* process is mainly used to treat polluted soils; thus, polluted soils are moved from the site of pollution to another location where it would undergo a microbial process to immobilise the metals responsible for the contamination. On the other hand, in the *in situ* procedure, the pollution is treated on-site (Cao et al. 2022).

Thus, microbial species (alone, in consortia, or in association with plants) emerge as useful tools in several biotechnological processes, including the bioremediation of contaminated environments. This is due to their unique metabolism (especially in the case of extremophilic microorganisms), their ability to use environmental contaminants as a source of nutrients and energy, and their small contact surface compared to plants and macroalgae (Jia et al. 2016; Cao et al. 2022; Wu et al. 2023). In this context, most of the successful research reported up to date has been conducted with microbial consortia to treat polluted soils aiming for the removal, bio-adsorption, or carbonated precipitation of organic and inorganic pollutants (Bhatt et al. 2021; Zheng et al. 2021; Ibrar et al. 2022; Zhang and Zhang 2022). The conclusion that can be obtained from all these works is that microbial-based technologies are sustainable interventions for greener ecosystem recovery, especially when aiming at the removal of compounds like hydrocarbons, xenobiotics, and other environmental pollutants induced by anthropogenic stressors. Several successful case studies have provided evidence for this paradigm including the putative assumption that the technology is eco-friendly, cost-effective, and shows a high tendency for total contaminants mineralisation into innocuous by-products (Nwankwegu et al. 2022; Skariyachan et al. 2022).

Research carried out all over the world during the last decade has shown that extremophilic microorganisms in general, and particularly several members of the archaea domain, can be of high interest for biotechnological purposes including bioremediation due to their extraordinary molecular adaptations to be alive under extreme environmental conditions (high temperature, high sun radiation, low nutrient availability, high fluctuations regarding oxygen availability, high ionic strength among other stressful conditions) and, therefore, their genuine metabolic capabilities (Jeong and Choi 2020). Archaea were first described as a group of single-celled prokaryotic microorganisms living in extremophilic environments characterised by extreme environmental parameters like high or very low temperatures (thermophiles vs psychrophiles), high salinity (halophiles), low or high pH (acidophiles, alkalophiles), or strict anoxia (Kochhar et al. 2022; De Lise et al. 2023). Thus, the chemical composition of proteins, RNA, or DNA from extremophilic organisms differs considerably from that of their non-extremophilic counterparts. The consequence of these extraordinary metabolic capabilities as well as their mechanisms supporting genetic plasticity make them good natural tools for designing circular economy-based processes aiming for industrial and biotechnological applications using wastes as raw materials (Capes et al. 2011; Jeong and Choi 2020; De Lise et al. 2023).

## Haloarchaea as promising tools for bioremediation

Haloarchaea are prokaryotic microorganisms belonging to the Archaea domain that require salt concentrations to be alive (Oren 2008). Although initially they were described as moderate or extremophilic halophiles inhabiting extreme ecosystems, the studies carried out up to date reveal that they are more abundant and widely spread than initially thought. Most of the haloarchaeal species are grouped into two families (Halobacteriaceae and Haloferacaceae) and constitute the major microbial populations in salty environments (total salt concentrations above 20–25% w/v) (Gupta et al. 2015; Valentine 2007). These microorganisms inhabit ecosystems such as submarine brine pools, brine pockets within sea ice, natural salty lakes and lagoons, and salt marshes created by human beings. These environments are typically highly alkaline and can serve as sources of sodium chloride for human consumption (Oren 2008). In these ecosystems, haloarchaea constitute the major microbial populations.

From a metabolic point of view, several species can grow under microaerobic or anaerobic conditions. Regarding anaerobic metabolism, compounds such as oxychloride, acetate, and sulphur or nitrogenous compounds like nitrate or nitrate can be used as final electron acceptors instead of oxygen (Sorokin et al. 2016; Miralles-Robledillo et al. 2021). Several of the compounds mentioned can be toxic to living organisms depending on their concentration and specific chemical properties. However, microorganisms play a crucial role in environmental detoxification through their respiratory processes under microaerobic or anaerobic conditions, facilitating the removal of these compounds from soil or saline water. Thus, several species have been revealed as good candidates for biotechnological applications by both, using whole cells or their metabolites (Oren 2010; Martínez et al. 2022).

Currently, there is an increasing interest in the optimisation of bioremediation approaches in high-salt environments, which are mostly influenced by the discharge of industrial effluents. This interest is also increasing in the following:

- Countries in which pollution is increasing in combination with the extension of arid and semiarid regions because of climate change (North Africa, India, Middle East, etc.) (see references displayed in Table 1).
- Wastewater treatment-based companies looking for methods and strategies to recycle brines obtained after chemical and biological treatments (Oren 2010)

**Table 1:** Metabolic capabilities of haloarchaea of interest for the design of bioremediation approaches to remove pollutants

Pollutant/heavy metal	Species	Reference
Cadmium	*Haloferax mediterranei*	Saez-Zamacona et al. (2023)
Copper	*Haloferax mediterranei*	Llorca and Martínez-Espinosa (2022)
Silver	*Haloferax alexandrinus* *Haloferax lucentense*	Buda et al. (2023); Moopantakath et al. (2022)
Nitrogenous compounds (nitrate, nitrite, nitrosamines, etc.)	Haloarchaea*	Bernabeu et al. (2021); Torregrosa-Crespo et al. (2019)
Hydrocarbons/long-chain aliphatic alkane	*Halorientalis hydrocarbonoclasticus* Haloarchaea*	Bonfá et al. (2011); Kumar et al. (2020); Mukherji et al. (2020); Zhao et al. (2017)
Uranium	Haloarchaea*	Bader et al. (2018); Hilpmann et al. (2022)
Oil removal	Haloarchaea*	Al-Mailem et al. (2012; 2017)
Phenols	Haloarchaea*	Piubeli et al. (2014)
Mercury	Haloarchaea*	Al-Mailem et al. (2011)
Oxychlorates	*Haloferax mediterranei*	Martínez-Espinosa et al. (2015)
Azo dyes	Haloarchaea*	Kiadehi et al. (2018)
DMSO/sulfoxides	Haloarchaea*	Miralles-Robledillo et al. (2019); Sorokin et al. (2021a); Sorokin et al. (2021b)

Haloarchaea*: the study involves several species mainly belonging to the Halobacteriaceae and Haloferacaceae families

Thus, the increase of salinity and pollutants in soils and ground waters during the last years has indirectly reinforced attention in the search for microbial physiological and molecular mechanisms involved in salt-stress tolerance concomitantly with pollutant degradation, assimilation, and/or removal. However, the most used microbiological processes in biotechnology are not capable of being executed at high salt concentrations, indicating that microbial bioremediation of hypersaline-produced water requires halophiles or extreme halophiles (Oren 2010; Li et al. 2022).

In this context, haloarchaea have been successfully tested for biotechnological applications throughout the last decade (Martínez et al. 2022; Singh and Singh 2017). Among these applications, the following can be highlighted:

i) Synthesis of pigments showing high antioxidant activity. Although some haloarchaea can synthesise different types of carotenoids, the major pigment produced by the cells is the rare carotenoids called bacterioruberin (C50-type carotenoid) (Martínez et al. 2022).
ii) Synthesis of biopolymers of plastic nature. Several species have been described as efficient polyhydroxyalkanoate producers (PHAs), including the capability of producing PHBV-type biopolymer, which is one of the most marketed bioplastics due to its physicochemical properties (Oren 2010; Martínez et al. 2022).
iii) Synthesis of enzymes of interesting catalytic properties able to be stable and active at high temperatures. Most of these enzymes are resistant to denaturing agents such as detergents, organic solvents, and stable at extreme pH values (Oren 2008, 2010; Singh and Singh 2017; Martínez et al. 2022). Moreover, their proteins are rich in acidic amino acids, which allows the maintenance of stable conformation and activity at high salt concentrations (Oren 2008).

**Fig. 1:**
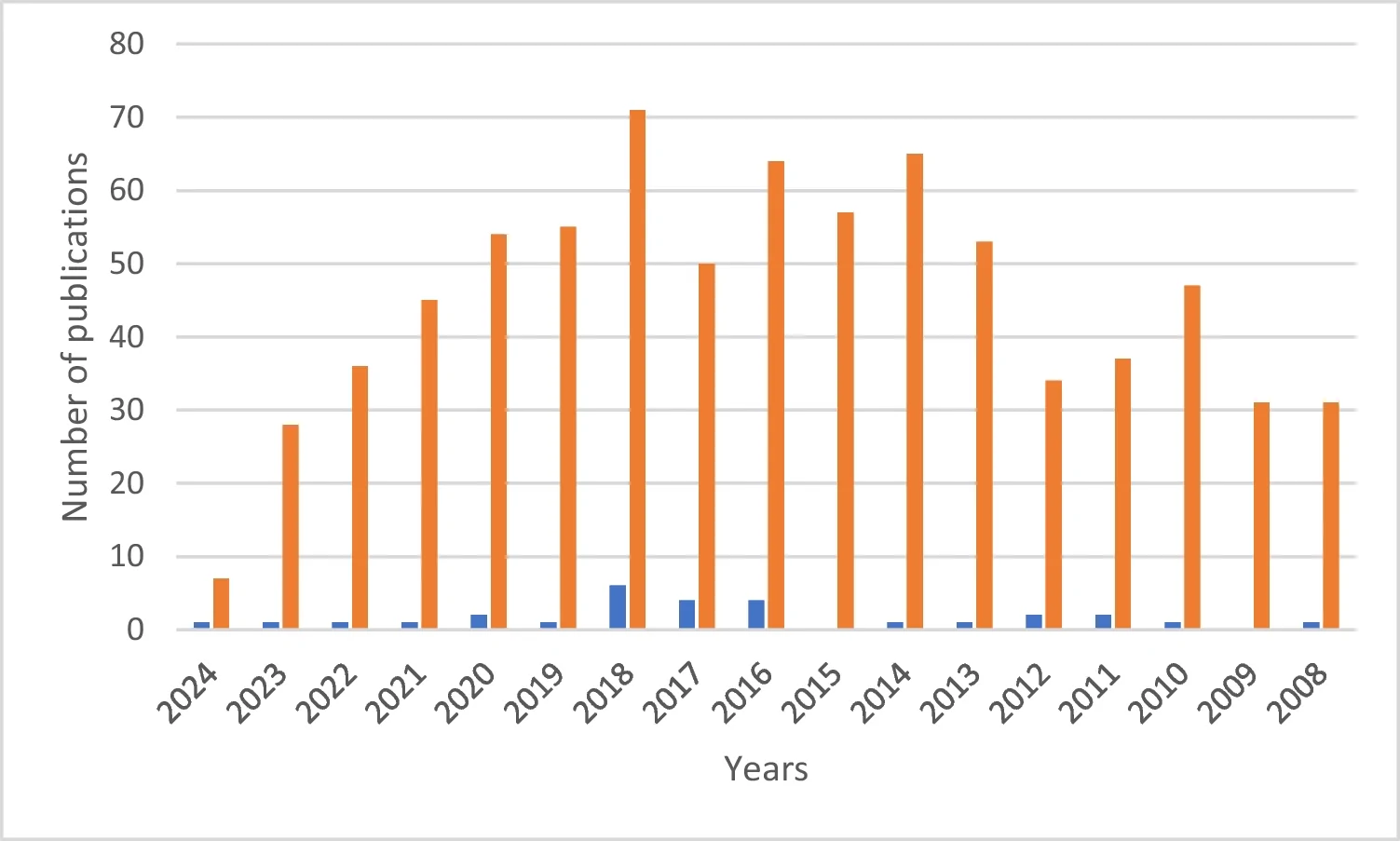
Number of publications identified through PubMed focused on the use of archaea (archaea AND bioremediation: orange colour) and haloarchaea (haloarchaea AND bioremediation: blue colour) as model organisms in bioremediation approaches (source PubMed, accessed on 17 May 2024). In the case of archaea, only data from the years that coincide with publications of studies with haloarchaea have been presented

Biochemical pathways related to most of the main biogeochemical cycles (like the iron, sulphur, or nitrogen cycles), heavy metals, aromatic compounds, or hydrocarbons have been described in archaea as potentially useful for biotechnological applications due to their catalytic efficiency. Based on databases, around 900 publications have been reported since 1978 demonstrating applications of various species of archaea in bioremediation (in all its variants) of water, soils, or sludge (Fig. 1). These studies cover from a variety of points of view (biochemistry, genetics, omics sciences, molecular engineering, system biology, etc.), all the benefits and disadvantages that the use of archaeal species in bioremediation would have, both on a small and large scale, as well as in situ and ex situ approaches (https://pubmed.ncbi.nlm.nih.gov/?term=archaea%20and%20bioremediation&sort=date&page=3). Only around 30 of them involved haloarchaeal species as model organisms for bioremediation proposals in saline and hypersaline wastewater treatments, thanks to their high tolerance to salt, metals, and organic pollutants (Oren 2010; Singh and Singh 2017; Martínez et al. 2022). These investigations focused on haloarchaea have been mainly conducted in the last decade as displayed in Fig. 1; therefore, this field of study can be considered innovative and promising.

The bibliometric analysis of the references identified as explained in Fig. 1 remarks the abundance of publications in countries like the USA followed by China, India, Kuwait, and Spain. Considering that the USA, China, India, and the European Union are among the most polluted areas in the world and that several areas of India, Spain, and the Middle Eastern countries are mainly characterised by hypersaline environments, it can be concluded that this recent research area is mainly supported by research groups directly facing the problems related to environment pollution and the expansion of extremophilic environments due to climate change in the geographical context where they are developing the work. So far, mesophilic microorganisms have successfully been used for wastewater treatment, and many of these processes produce brines that cannot be further treated biologically by mesophilic microbes due to their low tolerance to salt. Thus, haloarchaea are revealed as powerful tools to complete wastewater treatments including the management of the final brine produced. Table 1 summarises the main applications described up to date regarding the metabolic capabilities of haloarchaea that could be of interest in the bioremediation of salty soils and water.

Among all the haloarchaeal species tested for bioremediation, species belonging to the *Haloferax* genus, particularly *Haloferax mediterranei*, are probably the best characterised and revealed as good models for viable bioremediation applications. *H. mediterranei* is a haloarchaeon from the family Haloferacaceae (Gupta et al. 2015), firstly isolated from brines taken from seawater evaporation ponds located in the municipality of Santa Pola (near Alicante, Spain) (Rodríguez-Valera et al. 1980). This haloarchaeon can grow aerobically, microaerobically, and anaerobically in a broad range of NaCl concentrations ranging from 1.0 to 5.2 M (Gupta et al. 2015; Martínez et al. 2022; Rodríguez-Valera et al. 1980; Torregrosa-Crespo et al. 2019), thanks to its efficient metabolism and genome stability at moderate and high salt concentrations (Capes et al. 2011).

As can be concluded from the works listed in Table 1, *H. mediterranei* can remove most of the nitrogen compounds present in brines and soil (including nitrate, nitrite, and ammonium), specifically under anoxic conditions after the induction of the denitrification pathway (*H. mediterranei* shows a complete denitrifier phenotype) (Torregrosa-Crespo et al. 2019; Bernabeu et al. 2021). This is particularly interesting in the case of nitrate and nitrite which are highly toxic oxyanions for most living beings (nitrite for instance causes gastric cancer in human beings). Besides, this species can use nitrate and nitrite not only through denitrification but also as sole nitrogen for growth, thanks to the pathway termed assimilatory nitrate reduction. Considering both pathways, *H. mediterranei* can remove up to 2 M of nitrate and up to 50 mM of nitrite apart from ammonium from brines (Torregrosa-Crespo et al. 2019; Bernabeu et al. 2021; Miralles-Robledillo et al. 2021). In principle, the concentrations of nitrogenous compounds in natural brines of saltern ponds and marshes are relatively low or even insignificant. However, their proximity to vast extensions of lands used for agriculture, where there is excessive use of fertilisers, is leading to the appearance of concentrations of nitrate, nitrite, and ammonium above the thresholds established by current legislation in Europe.

Apart from the removal of nitrate and nitrite from water and brines, thanks to denitrifying haloarchaea like *H. mediterranei*, the enzyme termed “respiratory nitrate reductase” (catalysing the first reaction in the denitrification pathway) can efficiently reduce bromate and (per)chlorate, thanks to the use of these compounds as terminal electron acceptors under anoxic conditions (Martínez-Espinosa et al. 2015). (Per)chlorates are a by-product of chlorine-based products that can be found as a disinfectant in the food processing industry, in processes aiming for the purification of drinking water; as part of herbicides, and pesticide formulas; or as a component of fireworks among other uses (Martínez-Espinosa et al. 2015). In a 2015 study, the European Food Safety Authority (EFSA) found that the current level of chlorate that people get through food and drinking water is too high, and connections between (per)chlorate ingestion and health concerns for at-risk groups were connected at that time (Martínez-Espinosa et al. 2015; https://www.efsa.europa.eu/en/efsajournal/pub/4135). For this reason, it is important to decrease the use of oxychlorides and remove them from the environment. Here, haloarchaea are powerful biological tools because of their high capability to transform them into fewer toxic compounds (Martínez-Espinosa et al. 2015).

Other works recently demonstrated that *H. mediterranei* as well as other members of the haloarchaeal group have the necessary molecular machinery to grow in the presence of pollutants like crude oil, heavy metals, phenols, and hydrocarbons, thus resulting in the assimilation, modification, degradation, and even complete removal of those toxic compounds (Table 1). Regarding the removal of crude oil or phenols by haloarchaea, simple practises like in vitro haloarchaeal growth in culture media containing crude oil and supplemented with extra nitrogen sources or other essential ions like magnesium and potassium allowed the efficient removal of those toxic compounds. Besides, the incubation of the cells under continuous illumination lost double as much more oil than samples incubated in the dark. Other optimizations like the addition of vitamins to promote hydrocarbon removal by haloarchaea have been tested finding that the oil and pure hydrocarbon consumption potential of all haloarchaeal species monitored was enhanced by vitamin additions. The most effective vitamins were thiamin, pyridoxine, and vitamin B12 (Bonfá et al. 2011; Al-Mailem et al. 2012, 2017; Zhao et al. 2017).

It has also been described the capability of several haloarchaea to produce nanoparticles in the presence of metals. For example, *Haloferax alexandrinus* RK_AK2, *Haloferax lucentense* RK_MY6, *Halococcus salifodinae* BK3, and *Halococcus salifodinae* BK18 can make silver nanoparticles (Srivastava et al. 2013, 2014, 2015; Moopantakath et al. 2022; Buda et al. 2023). The production of gold particles as well as particles from metalloids like selenium has also been described from the *Haloferax* genus [60, 61]. Nanoparticles have been described as small particles showing sizes ranging between 1 and 100 nm (consequently, they are undetectable by the human eye). Nanoparticles can exhibit significantly different physical and chemical properties to their larger material counterparts, and currently, they are used in several industrial processes (manufacture of transparent sunscreens, scratchproof eyeglasses, crack-resistant paints, anti-graffiti coatings for walls, stain-repellent fabrics, self-cleaning windows, and ceramic coatings for solar cells, etc.) or biological/biomedicine approaches (as fluorescent biological labels, for drug and gene delivery, detection of pathogens (viruses or bacteria) and biomolecules of interest like proteins, tissue engineering, etc.). Considering the potential uses of nanoparticles and the current interest in them in biomedicine and pharmaceutics, haloarchaeal species could be successfully used as cell factories to naturally produce nanoparticles concomitantly with the removal of heavy metals (Martínez et al. 2022; Moopantakath et al. 2022; Joseph et al. 2023). Consequently, the capability of some haloarchaea to make nanoparticles in culture media containing metals makes it possible to design circular economy-based processes in which pollutants like metals and metalloids can be removed from salty soils, brines, and wastewaters by producing nanoparticles highly marketed.

## Challenges to face in the future to advance knowledge on this topic: some conclusions

Biologically based remediation processes are gaining importance instead of chemical or solid methods aiming at the treatment of polluted sites. Thus, bioremediation, biostimulation, and bioaugmentation involving microorganisms are suggested to be suitable alternatives, and due to this fact, they have received a great deal of attention in recent years. In this context, extremophilic microorganisms showing genuine metabolism and high catalytic capabilities may represent a good option for the bioremediation of contaminated sites. In some studies, wild-type strains of extremophilic organisms have been used as model organisms to explore bioremediation strategies. In other cases, incidents of highly toxic spills into water (rivers, seas, and oceans) or soil, resulting from accidents at nuclear power plants or the sinking of ships carrying oil or other chemical products, have been analysed in detail to find microbial strains capable of surviving under these stressful conditions. Some microbial strains, when subjected to these toxicity events, may have exponentially modified themselves to become more tolerant and/or resistant to initially toxic compounds. The goal is to use microorganisms with extraordinary metabolic capabilities as tools for bioremediation. Examples of the last topic can be found in the literature concerning bioremediation by microorganisms with spills of crude oil, phenols, nitrogen, thallium, and others.

Halophilic microorganisms, especially haloarchaea, represent a highly promising group of extremophilic microorganisms able to remove toxic compounds from soils and water, thanks to their effective metabolism under extreme conditions or even in the presence of highly toxic compounds. During the last few decades, several studies hypothesised the potential application of extremophilic microorganisms in biotechnology (enzymes of high catalytic profile, production of bioplastics, synthesis of pigments with significant antioxidant activity, small peptides showing antibacterial activities, etc.). The use of these microorganisms as components of bioremediation strategies aimed at treating contaminated sites has emerged as promising, thanks to the remarkable metabolic capabilities demonstrated by numerous extremophilic microorganisms. Regarding extreme halophiles (most of them haloarchaea), recent research has demonstrated that they can remove highly toxic compounds like phenols, nitrite, crude oil, heavy metals, and oxychlorides, thanks to their effective metabolism under extreme conditions. The research on the physiology, biochemistry, and molecular biology of haloarchaea will be a remarkable issue in biotechnological applications, especially in the bioremediation of naturally contaminated water (mostly salty water and brines) in the next few years. In this regard, the microbial strains traditionally used in wastewater treatments do not tolerate mid or high-salt concentrations. Given the significant worldwide impact of brine production as residues in various industrial processes (such as textile manufacturing, pickling, leather tanning), the exploration of the metabolic capabilities of haloarchaea as an alternative to other microbial strains represents one of the primary challenges to be thoroughly investigated in the near future. Those processes could be developed in bioreactors or even on land due to the high capacity of haloarchaeal to be adapted to extreme parameters or even dramatic changes in terms of temperature, light, or nutrient availability in daily and/or seasonal cycles.

The development of studies based on omics to improve the characterisation of haloarchaea as well as innovating methods to favour the growth of haloarchaea at mid and large scales (not only indoor but also outdoor) will contribute to the efficient use of those microorganisms, all this to elucidate the optimum range of conditions for suitable bioremediation applications as isolated processes or in combination with green technologies and circular economy-based processes.
